# Bias analysis applied to Agricultural Health Study publications to estimate non-random sources of uncertainty

**DOI:** 10.1186/1745-6673-2-15

**Published:** 2007-11-26

**Authors:** Timothy L Lash

**Affiliations:** 1 Department of Epidemiology, Boston University School of Public Health, 715 Albany St., TE3, Boston, MA, USA

## Abstract

**Background:**

The associations of pesticide exposure with disease outcomes are estimated without the benefit of a randomized design. For this reason and others, these studies are susceptible to systematic errors. I analyzed studies of the associations between alachlor and glyphosate exposure and cancer incidence, both derived from the Agricultural Health Study cohort, to quantify the bias and uncertainty potentially attributable to systematic error.

**Methods:**

For each study, I identified the prominent result and important sources of systematic error that might affect it. I assigned probability distributions to the bias parameters that allow quantification of the bias, drew a value at random from each assigned distribution, and calculated the estimate of effect adjusted for the biases. By repeating the draw and adjustment process over multiple iterations, I generated a frequency distribution of adjusted results, from which I obtained a point estimate and simulation interval. These methods were applied without access to the primary record-level dataset.

**Results:**

The conventional estimates of effect associating alachlor and glyphosate exposure with cancer incidence were likely biased away from the null and understated the uncertainty by quantifying only random error. For example, the conventional p-value for a test of trend in the alachlor study equaled 0.02, whereas fewer than 20% of the bias analysis iterations yielded a p-value of 0.02 or lower. Similarly, the conventional fully-adjusted result associating glyphosate exposure with multiple myleoma equaled 2.6 with 95% confidence interval of 0.7 to 9.4. The frequency distribution generated by the bias analysis yielded a median hazard ratio equal to 1.5 with 95% simulation interval of 0.4 to 8.9, which was 66% wider than the conventional interval.

**Conclusion:**

Bias analysis provides a more complete picture of true uncertainty than conventional frequentist statistical analysis accompanied by a qualitative description of study limitations. The latter approach is likely to lead to overconfidence regarding the potential for causal associations, whereas the former safeguards against such overinterpretations. Furthermore, such analyses, once programmed, allow rapid implementation of alternative assignments of probability distributions to the bias parameters, so elevate the plane of discussion regarding study bias from characterizing studies as "valid" or "invalid" to a critical and quantitative discussion of sources of uncertainty.

## Background

The Agricultural Health Study (AHS) enrolled 57,311 applicators licensed to apply restricted use pesticides into a large prospective cohort study between 1993 and 1997. Among the initial objectives was to identify and quantify cancer risks among men, women, whites, and minorities associated with direct exposure to pesticides and other agricultural agents [1]. Comprehensive information on exposure to 22 pesticides, use of personal protective equipment, candidate confounders, and demographic data were collected by baseline questionnaire at enrollment. A complete description of the initial methods has been published elsewhere [1].

This paper focuses on two publications from the AHS. First, Lee *et al*. (2004) published a study to "examine the cancer experience of alachlor applicators" [2]. Cohort members were matched to population-based cancer registry files in the two enrollment states (North Carolina and Iowa) for case identification and to state and national death registries to identify decedents. Cohort members who emigrated from the enrollment states were identified using records of the Internal Revenue Service, motor vehicle registries, and registries of pesticide applicators maintained by the state agricultural departments. Prevalent cancer cases and applicators with missing data on alachlor application were excluded, leaving 26,510 exposed applicators and 23,470 nonexposed applicators. Standardized incidence ratios were calculated for these two exposure groups for all cancers and sub-types with five or more exposed cases using incidence rates stratified by state, age, calendar-time, and race. The exposed group was divided into quartiles of cumulative alachlor exposure based on two exposure metrics: lifetime exposure-days and intensity-weighted exposure days. Dose-response analyses across exposure quartiles used Poisson regression to adjust for candidate confounders and tests of trend were made by the method of Breslow and Day [3]. Among alachlor-exposed applicators, the authors reported an increasing trend for incidence of all lymphohaematopoietic cancers associated with cumulative exposure. They also reported associations between the highest exposure to alachlor and leukemia occurrence (rate ratio = 2.83, 95% confidence interval (CI) 0.74, 10.9) and multiple myeloma occurrence (rate ratio = 5.66, 95% CI 0.70, 45.7).

Second, De Roos *et al*. (2005) published a study of "site-specific cancer incidence associated with glyphosate use among pesticide applicators in the Agricultural Health Study (AHS) cohort" [4]. AHS cohort members were matched through 2001 to population-based cancer registry files in the two enrollment states for case identification and to state and national death registries to identify decedents. The median follow-up time equaled 6.7 years. Prevalent cancer cases (n = 1074) and applicators with missing data on glyphosate application (n = 1678), age (n = 7), or who did not contribute person-time (n = 298) were excluded, leaving 40,376 applicators who ever used glyphosate and 13,280 who never used glyphosate. The exposed group was divided into tertiles of cumulative glyphosate exposure based on two exposure metrics: lifetime cumulative exposure-days and lifetime cumulative intensity-weighted exposure days. Poisson regression was used to estimate the rate ratio and 95% confidence interval for cancers with thirty or more incident cases. Rate ratios were calculated to compare ever-users with never-users, as well as to assess dose-response as a function of the median of tertile, quartile, and quintile of cumulative glyphosate exposure. In some analyses, never users provided the reference condition and in other analyses the group with the lowest cumulative exposure provided the reference condition. Glyphosate exposure was not substantially related to cancer incidence, except possibly for multiple myeloma (adjusted rate ratio = 2.6, 95% CI 0.7, 9.4).

Both of these publications provided an estimate of the effect of exposure to pesticide on the occurrence of cancer or cancers, and these estimates derive from epidemiologic study designs without the benefits of randomization. Epidemiologic research is an exercise in measurement, with an overarching objective of obtaining a valid and precise estimate of either the occurrence of disease in a population or the effect of an exposure on the occurrence of disease. Conventionally, epidemiologists present their measurements in three parts: a point estimate (e.g., a rate ratio), a frequentist statistical assessment of the uncertainty (e.g., a confidence interval, but also sometimes a p-value), and a qualitative description of the threats to the study's validity. Without randomization of study subjects to exposure groups, however, the point estimates, confidence intervals, and p-values lack their optimal frequentist interpretations [5].

Randomization and a hypothesis about the expected allocation of outcomes – such as the null hypothesis – allow the observed result to be compared with a probability distribution to estimate the probability of the observed association, or associations more extreme, under the null hypothesis. This comparison provides an important aid to causal inference [5]. When the exposure is allocated by non-random influences, such as allocation to occupations that entail exposure to pesticides, the comparison provides a probability that the variation in the outcome distribution is attributable to chance, under the null hypothesis, as opposed to the combined effects of exposure and systematic errors. Causal inference resting on an observed association therefore requires speculation about the strength of the systematic errors compared with the strength of the exposure effects.

This speculation can be accomplished quantitatively by Monte Carlo simulation [6-8] or other methods [9], and is then called a bias analysis or a sensitivity analysis. The conventional approach offers no such quantification, and instead conducts the speculation qualitatively by describing the study's limitations. This approach to analysis of systematic error will frequently fail to safeguard against tendencies to favor exposure effects over systematic errors as an explanation for observed associations [10]. Bias analysis has the potential to safeguard against these failures, and is most valuable when studies yield narrow conventional confidence intervals – so have little residual random error – and when these studies are susceptible to a limited number of systematic errors. Such studies often appear to be an adequate basis for inference or policy action, even though only random error has been quantified by the conventional confidence interval. Quantification of the error due to the limited number of biases will safeguard against inference or policy action that takes account of only random error. Without a quantitative assessment of the second important source of error – systematic error – the inference or policy action would be premature. The alachlor and glyphosate publications provide examples for which bias analysis may be valuable.

## Methods

### Introduction to Monte Carlo bias analysis

I used Monte Carlo simulation to assess the bias and uncertainty introduced by systematic errors in the alachlor and glyphosate studies. I have previously described a general strategy for modifying data sets by simulation to account for systematic errors [11,12]. The general strategy begins with identification of the important sources of systematic error likely affecting the estimate of association. The general strategy then continues with the bias analysis, which is a modification of the observed data set using estimates of bias parameters and then estimation of the association using that modified data set. Bias parameters are assigned probability distributions, and then sampled from the assigned distribution over many iterations. The frequency distribution of revised estimates of association yields an estimate of central tendency (the median, equivalent to the point estimate of a conventional analysis) and a simulation interval (*e.g*., the 2.5% and 97.5% are equivalent to the limits of a conventional frequentist confidence interval).

### Bias analysis of the alachlor paper

#### Identification of important sources of systematic error

The potential for inference regarding a causal relation between exposure to alachlor and lymphohematopoietic cancers rests primarily on the dose-response analysis. Without access to primary data, and without the reported person-time in each dose-category, only a limited bias analysis could be implemented. The bias analysis takes account of only two sources of uncertainty: (1) the uncertainty in the total number of cases, which will be assessed by applying reasonable estimates of the positive and negative predictive values, and (2) the uncertainty in the exposure assignment, which will be modeled by (a) selecting exposure dose-points from triangular distributions centered on the categories' midpoints, (b) reallocating cases to exposure categories by drawing them from a multinomial distribution centered on the observed proportions, and (c) selecting rate ratios from their reported log-normal distributions.

#### Modeling uncertainty in the number of incident cancer cases

Matching to state cancer registries identified incident cancer cases. Cohort members who matched registry records were assigned a cancer diagnosis according to the 9^th ^revision of the International Classification of Disease (ICD-9). The investigators provide no information on the identifying variables and algorithms that were used to match cohort members to the cancer registries or on the quality of the state cancer registry data. An investigation of the quality of the matching algorithm and completeness of the state registry data would provide bias parameters that would allow quantitative assessment of the sensitivity of the study findings to these sources of uncertainty [13]. However, without the original data with which to assess the quality of the match, I will use assumptions about the bias parameters. I assigned a trapezoidal distribution to the positive predictive value with minimum = 0.95, maximum = 1.0, and modes of 0.98 and 0.99. For each iteration of the bias analysis, a predictive value was drawn from this distribution to represent the probability that a case in the analysis was a true case. Similarly, I assigned a fixed negative predictive value equal to 0.99, which represents 99% probability that noncases were, in fact, not diseased. This negative predictive value was applied in each iteration such that no more than 10% of the total observed cases could be added to the analysis. In most cases, no additional cases were added, reflecting the assumption that false-negative cases were very rare.

#### Modeling classification of dose used in the dose-response analysis

The authors presented a dose-response analysis that compared rates in the second, third, and fourth quartiles of cumulative exposure to the rate in the first quartile. Comparisons are ratio measures with accompanying 95% confidence intervals. Cohort members were originally split into three groups: (a) those with missing data on application of alachlor, (b) those who reported any application of alachlor, and (c) those who reported no application of alachlor. The group with missing data was excluded from further analysis.

The authors did not address the quality of the exposure information, although it is certainly measured with substantial uncertainty. For example, Blair et al (2002) reported agreement between 79% and 87% for ever-use of a subset of pesticides in a subsample of 4088 Iowa AHS applicators [14]. Agreement was lower (typically 50% to 60%) for measures that involved duration, frequency, or decade of first use of specific pesticides, which are variables that would enter into calculations of cumulative exposure. Sensitivity and specificity or predictive values would be more valid bias parameters that could be used in a quantitative assessment of uncertainty arising from errors in exposure classification. However, with no true gold standard, a measure of intra-rater reliability (similar to agreement) for exposed and non-exposed will have to suffice and has been used in previous bias analysis [15]. To assess the error from misclassification of dose, I assigned triangular probability distributions to each of the dose-categories. The minimum and maximum parameters of the triangular distributions equaled the reported bounds of the exposure categories and the mode of the triangular distributions equaled the midpoints of the range of the exposure categories. The authors used an open-ended dose-category to depict the highest dose group (>116.1 lifetime alachlor exposure-days), although they must have assigned a dose-point to this category to implement their dose-response analysis method. The unknown dose-point presented a barrier to replicating the dose-response analysis, since I had to impute it from the available data. I assigned an imputed dose-point (144.4 lifetime alachlor exposure-days) as the mode and 200 lifetime alachlor exposure-days as the maximum.

I also reallocated the total number of cases (modeled after application of the predictive values defined above) to exposure categories by drawing their distribution from a multinomial distribution centered on the proportion of cases in each exposure category. I drew from the multinomial following the direct method described by Davis [16]. Finally, I drew rate ratios from normal distributions centered on the log of the observed rate ratio reported for each exposure category and with standard error equal to the variance calculated from the reported 95% confidence intervals in each exposure category. This strategy underestimates the uncertainty in each of these rate ratios, since it assumes they were influenced by no systematic error.

#### Quantitative Analysis

The authors used an appropriate method to assess the dose-response trend [3]. To replicate the method, both the number of cases and accrued person-time in each dose group would be required. The authors provide the number of cases in dose-groups, but nowhere provide the accrued person-time. To overcome this barrier, I used the method of Rothman and Boice to assess the dose-response trend [17], which requires the number of cases and the observed rate ratios in each dose category, but does not require person-time. To model the uncertainty, I drew a value from each of the respective distributions and reassessed the dose-response trend using the method of Rothman and Boice [17]. This reassessment yielded a p-value (a measure of the strength of association as assessed by the trend) and a measure of the bias in the original trend (original slope divided by iteration's slope). This process was repeated for ten thousand iterations and the p-value and bias were recorded for each iteration. The cumulative frequency distribution of the p-value and bias inform the inference that can be made from the bias analysis.

### Bias analysis of the glyphosate paper

#### Identification of important sources of systematic error

Table two of the publication [4] showed the rate ratios comparing cancer incidence rates for ever-users of glyphosate with never-users of glyphosate. Thirty-two cases of multiple myeloma occurred. The rate ratio comparing ever-use with never-use adjusted for only age equaled 1.1 (95% CI 0.5–2.4), whereas the rate ratio adjusted for age, demographic and lifestyle factors, and other pesticide use equaled 2.6 (95% CI 0.7–9.4). This second adjusted estimate of effect inspired a substantial amount of subsequent analysis and inference, so merits careful inspection. As with the alachlor paper, without access to primary data, only a limited bias analysis could be implemented.

The change in the rate ratio from 1.1 when adjusted only for age to 2.6 when adjusted for the additional factors suggests a substantial amount of confounding controlled by the adjustment or a substantial amount of bias introduced by the adjustment (or some combination of the two). The relative risk due to confounding is a measure of the direction and magnitude of confounding controlled by adjustment variables. In this case, the relative risk due to confounding by the additional adjustment variables would have to equal 2.36 to explain the change in the rate ratio from the age-adjusted estimate of 1.1 to the fully adjusted estimate of 2.6. The relative risk due to confounding is bounded by the following limits: (a) the odds ratio associating the exposure with the confounder [OR_EC_], (b) the odds ratio associating the disease with the confounders [OR_DC_], (c) the inverse of the prevalence of the confounder in the unexposed group [1/P_C + E-_], (d) OR_DC_/{(1-P_C + E-_) + OR_DC_*P_C + E-_}, and (e)OREC/{(1-P_C + E-_) + OR_EC_* P_C + E-_} [18].

I have assessed the potential for confounding by education to illustrate the value of bounding the relative risk due to confounding. The odds ratio associating glyphosate use with level of education (item a) equaled 1.94, the inverse of the prevalence of the confounder in the unexposed group equaled 1.46 (item c), and item (e) equaled 1.18. While I cannot estimate items b or d from above, I do know that the relative risk due to confounding cannot exceed the minimum of the three bounds that I can estimate (1.18). The observed relative risk due to confounding (2.36) is far in excess of that bound. None of the variables controlled after age, individually or jointly, would be expected to yield a relative risk due to confounding of the size observed (2.36), particularly conditional on the initial adjustment for age.

Indeed, the authors acknowledged that the change in estimate from 1.1 to 2.6 derived more from restricting the sample size to those without missing data on the adjustment variables than from the adjustment itself. They wrote in the discussion section:

*Table *1* shows that the 54,315 subjects were included in the age-adjusted models, whereas because of missing data for covariates, only 40,719 subjects were included in fully adjusted analyses. The association of glyphosate with myeloma differed between the two groups, even without adjustment for any covariates, with no association among the full group and a positive association among the more restricted group*.

**Table 1:** Summary of sources of error, bias parameters, and assigned distributions used in the two bias analyses

**Source of potential error**	**Bias parameters**	**Assigned distributions**
**Alachlor Bias Analysis**		

number of incident cancer cases	positive predictive value negative predictive value	trapezoidal distribution with minimum = 0.95, maximum = 1.0, and modes of 0.98 and 0.99 fixed at 0.99
mismeasurement of cumulative exposure	cumulative exposure value assigned to each category	triangular distributions with minimum and maximum equal to reported bounds of exposure category, and mode equal to its reported midpoint
allocation of cases to cumulative exposure categories	number of cases in each exposure category	multinomial centered on the number of cases observed in each category
rate ratios assigned to each exposure category	log normal distribution	mean equal to the log of the reported hazard ratio for each category and variance imputed from its 95% confidence interval

**Glyphosate Bias Analysis**		

confounding of the association by all adjustment variables	relative risk due to confounding	trapezoidal distribution with minimum of 1 (no confounding after adjustment for age), lower mode of 1.18 (bound due to confounding by education), upper mode of 1.39 (all variables but state), and maximum 1.56 (all variables, including state)
exposure misclassification	sensitivity and specificity of exposure classification	triangular distribution with minimum 0.79, maximum 0.87, mode 0.82 based on agreement proportions

Unfortunately, the authors did not provide the age-adjusted rate ratio comparing ever-users with never-users restricted to subjects who had complete data, although they apparently calculated that rate ratio. Had they provided it, the reader would be able to judge the extent to which the fully adjusted rate ratio derived from restricting the sample to those without missing data. The authors went on to say in the discussion section:

*The increased risk associated with glyphosate in adjusted analyses may be due to selection bias or could be due to a confounder or effect modifer that is more prevalent among this restricted subgroup and is unaccounted for in our analyses*.

The prevalence of ever-use of glyphosate was equivalent in the full and restricted samples, so the strength of association between the confounder and the exposure would have to be larger in the restricted data set than in the full data set. That circumstance would describe selection bias, not confounding. The effect modification explanation is also implausible. If all applicators had the effect modifier in the restricted sample, then the rate ratio among the full sample (1.1) would have to equal an inverse variance weighted average of the 2.6 rate ratio in the 75% of applicators who had the effect modifier and were included in the restricted analysis and another rate ratio in the 25% of applicators who did not have the effect modifier and were excluded from the restricted analysis. That other rate ratio would have had to equal an extremely unlikely protective association between glyphosate use and rate of multiple myeloma among those with this hypothetical effect modifier for the weighted average effect to be null. Other scenarios would be even less plausible. The best explanation is that restricting the data set to those with complete data introduced an important selection bias.

Misclassification of exposure to glyphosate was a second major source of systematic error, just as it was for the alachlor publication. As for the alachlor analysis, cohort members were originally split into three groups: (a) those with missing data on application of glyphosate, (b) those who reported any application of glyphosate, and (c) those who reported no application of glyphosate. The same concerns about the quality of classification of exposure apply to the glyphosate analysis as they did to the alachlor analysis.

There were 32 cases of multiple myeloma, 75% of whom reported ever using glyphosate. Among all subjects, 79% reported ever using glyphosate. To calculate the expected number of exposed cases and persons, I used reliability data from the Agricultural Health Study reported by Blair and colleagues [14]. Although reliability data are not as informative a measure of validity as true sensitivity or specificity, it can be used as a reasonable approximation, as illustrated with an example in the Blair paper and in our earlier work [15].

#### Modeling the relative risk due to confounding

Since the relative risk due to confounding exceeded its plausible maximum, I adjusted for the potentially confounding variables in the bias analysis by drawing the relative risk due to confounding from a trapezoidal distribution. I parameterized the trapezoidal distribution using the data in the publication to calculate the bounds on the relative risk due to confounding for each individual confounder. For each potential confounder, the bound on the relative risk due to confounding was ultimately derived from item e. The greatest of these is item e from the education variable, so if adjustment for all variables were attained by adjusting for just this variable, then the relative risk due to confounding would equal its item e (OR_EC_/{(1-P_C + E-_) + OR_EC_*P_C + E- _= 1.18). If the confounding impacts of all of the variables are completely independent, which would require them to be unassociated, then the relative risk due to confounding would equal the product of their individual bounds on the relative risks due to confounding, which equaled 1.56. State is unlikely to be related to risk of multiple myeloma, but disease risk is not available within levels of the confounders, so the association cannot be examined. Recalculating the independent relative risk due to confounding without including the contribution from enrollment state yields an expected relative risk due to confounding of 1.39. I parameterized the trapezoidal distribution of the relative risk due to confounding with a minimum of 1 (no confounding after adjustment for age), a lower mode of 1.18 (the bound on relative risk due to confounding by education), an upper mode of 1.39 (the independent relative risk due to confounding by all variables but state), and a maximum of 1.56 (the independent relative risk due to confounding by all variablen, including state).

#### Modeling exposure misclassification

Blair *et al*. reported agreement for report of exposure to eleven pesticides ranging from 0.79 to 0.87 [14], with a value of 0.82 for glyphosate. I used these agreement proportions as the minimum, maximum, and mode of a triangular distribution for both sensitivity and specificity of glyphosate exposure classification. For each iteration, one value was drawn from the distribution to represent the sensitivity of exposure classification and a second value was drawn independently from the distribution to represent the specificity of exposure classification.

The bias analysis required that I calculate the probability that a person classified as exposed was truly exposed and the probability that a person classified as unexposed was truly unexposed. These are predictive values (positive and negative, respectively), not sensitivity and specificity. To calculate the predictive values, I used the following equations relating predictive values to sensitivity and specificity:

PPVi=pispis+(1−pi)(1−t)NPVi=(1−pi)t(1−pi)t+pi(1−s)
 MathType@MTEF@5@5@+=feaafiart1ev1aaatCvAUfKttLearuWrP9MDH5MBPbIqV92AaeXatLxBI9gBaebbnrfifHhDYfgasaacPC6xNi=xI8qiVKYPFjYdHaVhbbf9v8qqaqFr0xc9vqFj0dXdbba91qpepeI8k8fiI+fsY=rqGqVepae9pg0db9vqaiVgFr0xfr=xfr=xc9adbaqaaeGacaGaaiaabeqaaeqabiWaaaGceaqabeaacqWGqbaucqWGqbaucqWGwbGvdaWgaaWcbaGaemyAaKgabeaakiabg2da9KqbaoaalaaabaGaemiCaa3aaSbaaeaacqWGPbqAaeqaaiabdohaZbqaaiabdchaWnaaBaaabaGaemyAaKgabeaacqWGZbWCcqGHRaWkdaqadaqaaiabigdaXiabgkHiTiabdchaWnaaBaaabaGaemyAaKgabeaaaiaawIcacaGLPaaadaqadaqaaiabigdaXiabgkHiTiabdsha0bGaayjkaiaawMcaaaaaaOqaaiabd6eaojabdcfaqjabdAfawnaaBaaaleaacqWGPbqAaeqaaOGaeyypa0tcfa4aaSaaaeaadaqadaqaaiabigdaXiabgkHiTiabdchaWnaaBaaabaGaemyAaKgabeaaaiaawIcacaGLPaaacqWG0baDaeaadaqadaqaaiabigdaXiabgkHiTiabdchaWnaaBaaabaGaemyAaKgabeaaaiaawIcacaGLPaaacqWG0baDcqGHRaWkcqWGWbaCdaWgaaqaaiabdMgaPbqabaWaaeWaaeaacqaIXaqmcqGHsislcqWGZbWCaiaawIcacaGLPaaaaaaaaaa@6666@

Where *PPV *and *NPV *are the positive and negative predictive values, *s *is the sensitivity of exposure classification, *t *is the specificity of exposure classification, *p *is the prevalence of glyphosate exposure, and *i *is an index for the four combinations of exposure status (glyphosate exposed and unexposed) with disease status (cases and persons at risk).

#### Quantitative Analysis

To conduct an iteration of the bias analysis, I drew a value of the sensitivity and a value of the specificity from the trapezoidal distributions, then calculated the positive and negative predictive values for cases and persons at risk, conditional on the observed prevalence of glyphosate exposure. I used a random binomial distribution to model the number of expected exposed cases and persons and the number of expected unexposed cases and persons. For example, I drew the number of expected exposed cases using the random binomial distribution with 24 trials (the number of observed exposed cases) and the positive predictive value for cases as the probability of a success. This procedure would yield between 0 and 24 exposed cases, and the difference between the number of modeled exposed cases and 24 would be reclassified as unexposed cases. Similarly, I drew the number of expected unexposed cases using the random binomial distribution with 8 trials (the number of observed unexposed cases) and the negative predictive value for cases as the probability of a success. This procedure would yield between 0 and 8 unexposed cases, and the difference between the number of modeled unexposed cases and 8 would be reclassified as exposed cases. The total number of exposed cases would equal the number modeled as correctly classified as exposed (out of 24) and the number modeled as incorrectly classified as unexposed (out of 8). I used a similar procedure to model the total number of persons at risk, but using that population size and the predictive values calculated using the prevalence of glyphosate exposure in the total population. I then calculated the modeled risk ratio and divided it by the crude risk ratio (24/40,376/8/13,280 = 0.99) to obtain an estimate of the bias due to misclassification.

To combine the bias analysis applied to account for confounding and misclassification, I multiplied the conventional age-adjusted hazard ratio reported in the paper (1.1), by each iteration's relative risk due to confounding and its misclassification bias to obtain the hazard ratio (*HR*_s_) simultaneously accounting for both threats to validity. To account for random error, I calculated the standard error of the hazard ratio (*SE*) using the exposed and unexposed case counts generated by the misclassification procedure, drew a random standard normal deviate (z), and then calculated

ln(*HR*_*R*_) = ln(*HR*_*s*_) - *z*·*SE*

to yield the natural logarithm of the hazard ratio incorporating random error as well as systematic error (*HR*_*R*_).

## Results and discussion

### Results of the alachlor bias analysis

**Figure 1:**
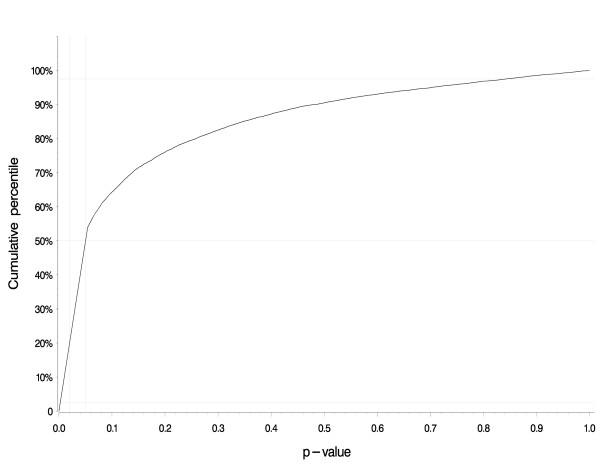
**Distribution of p-values yielded by 10,000 iterations of the bias analysis for alachlor**. The cumulative distribution of p-values from the 10,000 tests of trend generated by the bias analysis. This result pertains to the trend associating lifetime alachlor exposure-days with the rate of all lymphohematopoietic cancers. The p-value for this trend equaled 0.02 in the original paper, but fewer than 20% of the bias analysis iterations yielded a p-value of 0.02 or lower. Approximately 50% of the iterations yielded a p-value below 0.05 and the remainder yielded p-values greater than 0.05.

Figure 1 shows the cumulative distribution of p-values from the 10,000 tests of trend generated by the bias analysis. This result pertains to the trend associating lifetime alachlor exposure-days with the rate of all lymphohematopoietic cancers. The p-value for this trend equaled 0.02 in the original paper [2], but fewer than 20% of the bias analysis iterations yielded a p-value of 0.02 or lower. Approximately 50% of the iterations yielded a p-value below 0.05 and the remainder yielded p-values greater than 0.05.

**Figure 2:**
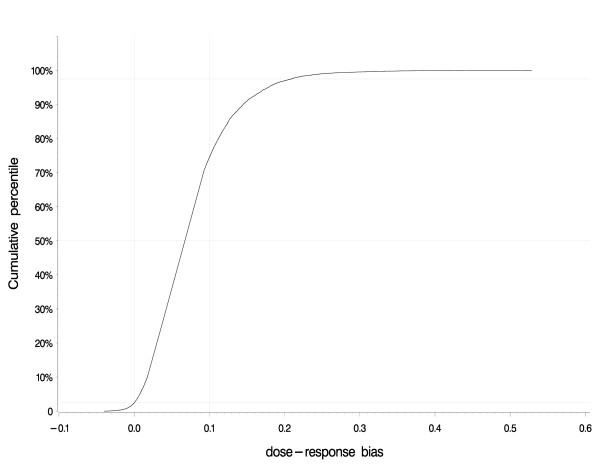
**Distribution of bias factors yielded by 10,000 iterations of the bias analysis for alachlor**. The cumulative distribution of the bias factor for the dose-response slope depicts the strength of association, separate from the precision of the slope. All results from the bias analysis yielded dose-response trends less strong than the original result. Approximately 2.5% of the dose-response trends yielded by the bias analysis equaled 0 (no trend) or below (reversed trend). Approximately 70% of the dose-response trends were less than 10% of the size of the original trend.

Figure 2 shows the cumulative distribution of the bias factor for the dose-response slope. The slope depicts the strength of association, separate from the precision of the slope. These two concepts are combined in the p-value. The cumulative distribution of bias factors shows that all results from the bias analysis yielded dose-response trends less strong than the original result. Approximately 2.5% of the dose-response trends yielded by the bias analysis equaled 0 (no trend) or below (reversed trend). Approximately 70% of the dose-response trends were less than 10% of the size of the original trend.

### Results of the glyphosate bias analysis

**Figure 3:**
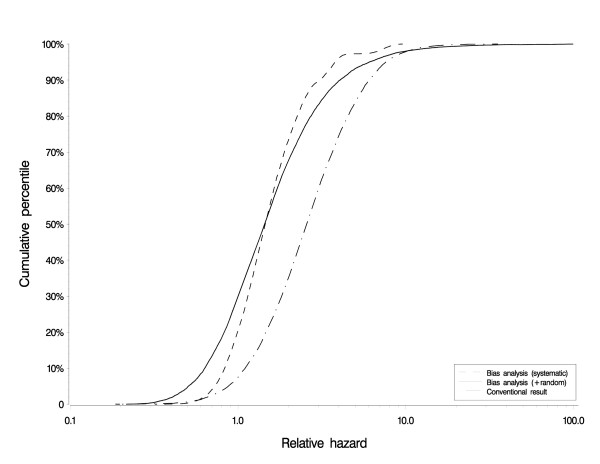
**Results of glyphosate bias analysis**. The stippled line shows the conventional fully-adjusted result, centered on a hazard ratio of 2.6 with 95% confidence interval of 0.7 to 9.4 associating ever-exposure to glyphosate with multiple myeloma. The solid line (random error bias analysis) shows the result of the bias analysis accounting for confounding and exposure misclassification, and incorporating random error. Its median equals 1.5 and its simulation interval was 0.4 to 8.9. The dashed line shows the bias analysis result without simultaneously incorporating random error. Its median equals 1.5, with 95% simulation interval from 0.65 to 6.3.

Figure 3 shows the overall results of the glyphosate bias analysis. The stippled line shows the conventional fully-adjusted result, centered on a hazard ratio of 2.6 with 95% confidence interval of 0.7 to 9.4. The solid line (random error bias analysis) shows the result of the bias analysis accounting for confounding and exposure misclassification, and incorporating random error. Its median equals 1.5, which suggests that the conventional result (2.6) was substantially biased, primarily by the adjustment for confounders. The adjustment for confounders influenced the result well beyond the confounding impact, probably by limiting the data set to those with complete data. The simulation interval about this complete bias analysis (0.4 to 8.9) was 66% wider than the conventional interval, showing that the conventional interval substantially understated the true uncertainty. The dashed line shows the bias analysis result without simultaneously incorporating random error, so depicts the additional uncertainty arising from systematic error. Its median equals 1.5, with 95% simulation interval from 0.65 to 6.3. This interval is 70% the width of the random error only conventional interval, further emphasizing that the conventional frequentist interval often substantially understates the true uncertainty of a result.

## Conclusion

When studies are designed without the benefit of randomized exposure assignment, conventional frequentist statistics underestimate the true uncertainty in an estimate of association. Despite qualitative descriptions of study limitations, the conventional frequentist statistics will often lead to overconfidence about the reported result and a tendency toward favoring the causal explanation for an association over bias explanations [10]. Bias analysis has been proposed as one solution to ameliorate this tendency.

I illustrate the value of bias analysis by applying it to two results reported from the AHS. Pesticide exposure is allocated by non-random influences, so studies of its association with health outcomes are candidates for bias analysis. Although both studies depicted the potential for systematic error in the description of limitations, neither applied a quantitative method to assess the additional uncertainty beyond random error contributed by these limitations. I implemented a bias analysis of the central findings from studies of the association of glyphosate exposure and alachlor exposure with cancer incidence. These bias analyses were conducted without access to record-level data, so relied entirely on published results, which illustrates that stakeholders can often address study limitations quantitatively even if original data are not available for analysis. Were record-level data available, additional bias analysis would have been possible, including further analysis of classification errors and an analysis of modeling error.

An advantage of bias analysis is that alternative assumptions about the parameterizations and bias models can be readily examined, and the results compared with one another. These comparisons provide a quantitative basis for disagreements among stakeholders about the potential impact of systematic error, and thereby remove the debate from the realm of qualitative criticism heavily influenced by politics and polemics. Instead, the analysis places the debate into the realm of quantitative analysis, in which differences between stakeholders can be readily traced to differences in the parameterization of the bias analysis, and the consistency of the different parameterizations with external information can be examined. These differences – when they yield substantially different results – provide fodder for discussion and may inspire additional research to characterize better the values to assign to the parameters. This approach to examining differences in interpretation of study results is far more consistent with the scientific enterprise, an inherently quantitative undertaking, than simple categorization of study results as "valid" or "invalid," since all studies are susceptible to imperfections.

Conditional on the validity of distributions assigned to the bias parameters, I showed that the central results from both studies were likely biased away from the null and that the conventional frequentist measures of error underestimated the true uncertainty. These methods of bias analysis are not difficult to implement and should be encouraged in future presentations of results.

## Competing interests

This project was supported, in part, by an unrestricted research grant from Crop Life America to the Boston University School of Public Health. The author has received $2000 in consulting fees from Monsanto Company, manufacturer of alachlor and glyphosate, in the past five years.

## Authors' contributions

TL conceived of the study, conducted the analyses, and wrote, read, and approved the final manuscript.
